# Thymoquinone Modulates Blood Coagulation *in Vitro* via Its Effects on Inflammatory and Coagulation Pathways

**DOI:** 10.3390/ijms17040474

**Published:** 2016-03-30

**Authors:** Vandhana Muralidharan-Chari, Jaehan Kim, Ahlam Abuawad, Mubeena Naeem, Huadong Cui, Shaker A. Mousa

**Affiliations:** The Pharmaceutical Research Institute, Albany College of Pharmacy and Health Sciences, 1 Discovery Drive, Rennselaer, NY 12144, USA; vandhana.mchari@acphs.edu (V.M.-C.); jaehan.kim@acphs.edu (J.K.); akabuawad@albany.edu (A.A.); mubeenanaeem@yahoo.com (M.N.); hdcui@yahoo.com (H.C.)

**Keywords:** cancer-associated thrombosis, complementary medicine, inflammation, natural supplement, thrombosis, thymoquinone

## Abstract

Thymoquinone (THQ) is a major component of black seeds. Given that both THQ and black seeds exhibit anti-cancer and anti-inflammatory activities, we hypothesized that THQ will affect cancer-associated thrombosis (CAT), which is primarily triggered by tissue factor (TF) and inflammation. The effect of both black seed-extracted and purchased (“pure”) THQ on normal blood coagulation was tested with *in vitro* thromboelastography (TEG) and activated partial thromboplastin time (aPTT) coagulation assays. The effect of pure THQ on CAT was tested with aPTT assay using pancreatic cancer cell lines that are either positive or negative for TF, and with TEG assay using lipopolysaccharide as an inflammatory trigger. Additionally, the direct effect of THQ on the inactivation of factors IIa and Xa was assessed. Since TNF-α facilitates crosstalk between inflammation and thrombosis by triggering the NF-κB pathway, we tested THQ’s ability to interfere with this communication with a luciferase assay. Both extracted and pure THQ had minimal effects on normal blood coagulation. Pure THQ reversed CAT initiated by both TF and inflammation to basal levels (*p* < 0.001). Mechanistically, while THQ had minimal to no effect on factor IIa and Xa inactivation, it strongly reduced the effects of TNF-α on NF-κB elements (*p* < 0.001). THQ has a minimal effect on basal coagulation and can reverse CAT *in vitro*, possibly by interfering with the crosstalk between inflammation and coagulation. This study suggests the utility of THQ as a preventative anticoagulant and/or as a supplement to existing chemotherapies and anticoagulant therapies.

## 1. Introduction

Thymoquinone (THQ) is the predominant bioactive compound derived from the oil phase of black seed, also known as black cumin (*Nigella sativa*) in the family Ranunculaceae. This is an annual herbaceous plant, native to Mediterranean countries, Pakistan, and India [1], and has been used in traditional Arab herbal medicine for the treatment of arthritis, lung diseases, and hypercholesterolemia [2]. THQ has been extracted from *Nigella sativa* seeds and investigated for therapeutic effects in various diseases including sepsis, inflammation, cancer, diabetes, and depression [3,4,5,6,7,8]. The anti-oxidant and anti-inflammatory effects of THQ and its ability to produce reactive oxygen species have been implicated in THQ’s prevention of chemical-induced carcinogenesis [9,10]. Several *in vivo* and *in vitro* studies provide evidence that THQ can prevent tumorigenesis by affecting stages in tumor progression, and this has been discussed in detail in recent reviews [3,11,12,13,14].

THQ exhibits anti-proliferative effects specifically on various malignant cells (human breast adenocarcinoma, glioblastoma, leukemia, lung cancer, colorectal carcinoma, pancreatic cancer, osteosarcoma, and prostate cancer), but showed little effect on non-cancerous cells, such as mouse fibroblasts and human normal lung fibroblasts and intestinal cells, suggesting that THQ can target proliferation in cancer cells while having limited effects on normal cells [15,16,17,18,19,20,21,22,23]. This property of anti-proliferative effects on cancer cells appears to occur either by inducing cell cycle arrest or by promoting cell death [24]. Combining THQ with conventional chemotherapeutic agents, such as gemcitabine on pancreatic cancer cells and cisplatin on non-small cell lung cancer cells, resulted in an increased anti-cancer effect, suggesting the possibility that THQ can be used in complement with conventional medicine to attain a greater therapeutic effect [19,22].

THQ has demonstrated anti-cancer effects by inhibiting tumor angiogenesis [25]. THQ inhibited human umbilical vein endothelial cell (HUVEC) migration, invasion, proliferation, and tube formation by down-regulating AKT/ERK signaling pathways. At low dosage, THQ also blocked tumor angiogenesis in a xenograft model of prostate cancer and prevented the growth of the human prostate tumor [25]. Thus, THQ exhibits anti-cancer effects by modulating tumor cell proliferation, cell death, tumor angiogenesis, and drug resistance in tumor cells. However, the role of THQ in tumor-associated thrombosis has not been investigated.

Recent studies indicate that thrombotic events in cancer patients are the second leading cause for cancer-related fatality after cancer itself, in addition to contributing to a poor prognosis for both short-term and long-term survival [26,27,28]. Venous events of cancer-associated thrombosis include deep vein thrombosis and pulmonary embolism which, along with visceral vein thrombosis, are described as venous thromboembolism (VTE). Arterial events associated with cancer-related thrombosis include stroke and myocardial infarction [29]. Local activation of coagulation has been demonstrated by the presence of fibrin deposition and platelet aggregation in and around various tumors by histopathological analyses [30,31]. These changes are reflected as shortened activated partial thromboplastic time (aPTT), increased blood levels of blood coagulation proteins, thrombocytosis, and increased concentrations of fibrin/fibrinogen degradation products [32].

Thrombus formation and blood coagulation processes in cancer occur via various mechanisms that are generally related to the host response to cancer. These mechanisms include activation of the coagulation cascades, fibrinolytic systems, acute phase reaction, inflammation resulting in cytokine production, and necrosis [33,34,35]. Although part of these phenomena can be explained by inflammatory responses associated with tumorigenesis, various studies have indicated the significance of the increased levels of tissue factor (TF) either on the tumor cells or on tiny particles, called microparticles, released by tumor cells and TF’s participation as a central trigger of the coagulation cascade [35,36,37]. Since THQ exhibits global anti-cancer effects via its anti-inflammatory effects or via reactive oxygen species production, we hypothesize that THQ can modulate blood coagulation and can reverse the coagulation mediated by cancer cells.

Using both black seed-extracted-THQ and commercially available pure THQ, we provide evidence for the first time that THQ possesses anticoagulant activity, as determined by *in vitro* coagulation assays. Both pure THQ and black seed-extracted-THQ modulate normal blood coagulation with minimal effect in a narrow dose range. In contrast, pure THQ can effectively reverse coagulation to basal levels when triggered by TF present on pancreatic cancer cells and by lipopolysaccharide (LPS). We also performed mechanistic studies and confirm that THQ decreases blood coagulation directly by decreasing factor Xa inactivation in blood coagulation cascade and by interfering with the crosstalk between inflammation and thrombosis.

## 2. Results

### 2.1. THQ in Black Seed Oil Extract Exhibits Anticoagulant Activity

**Figure 1:**
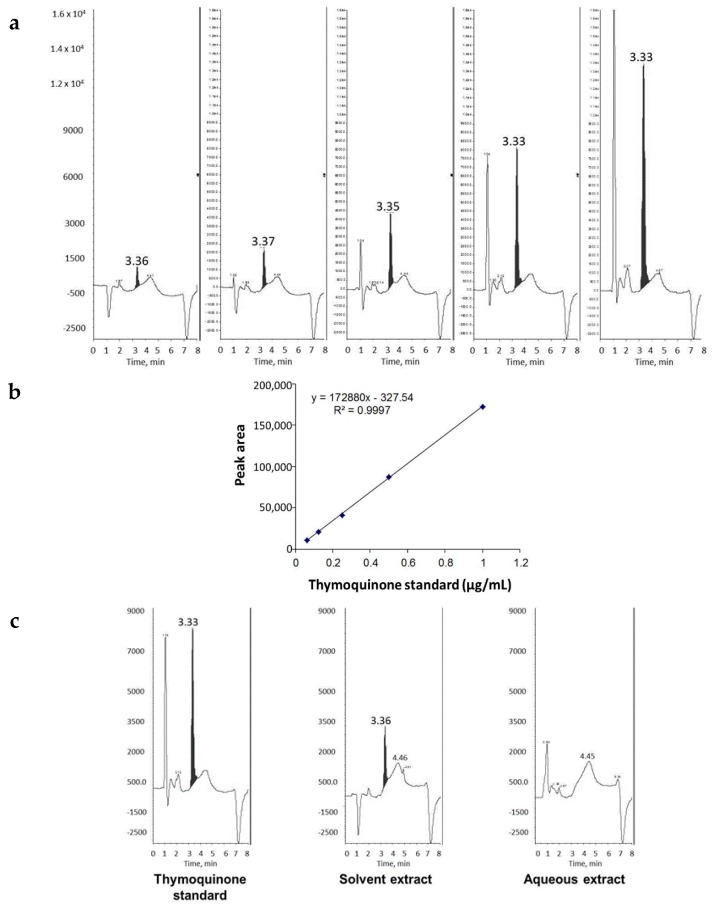
Detection and quantitation of THQ extracted from black seeds. (**a**) HPLC analysis of pure THQ standards in increasing concentrations (left to right); (**b**) calibration curve for pure THQ. The graph shows good linearity, indicating the power of THQ detection and quantitation by HPLC analysis; and (**c**) HPLC detection of THQ in solvent extract and aqueous extract of black seeds in comparison to THQ standard. Note the absence of THQ in aqueous extract.

The presence of THQ in the oil extract of black seeds was confirmed by comparing its retention time with that of pure THQ (~3.3 min, Figure 1a). The amount of THQ in the oil fraction was estimated against the calibration curve obtained from pure THQ, which showed good linearity in the range of 0.0625–1.0 µg/mL (Figure 1b). The absence of THQ in the aqueous extract was confirmed by HPLC analysis (Figure 1c). Two batches of black seeds were extracted with the solvent and THQ was estimated to be 1.6 mg/mL in both extractions by HPLC.

**Figure 2:**
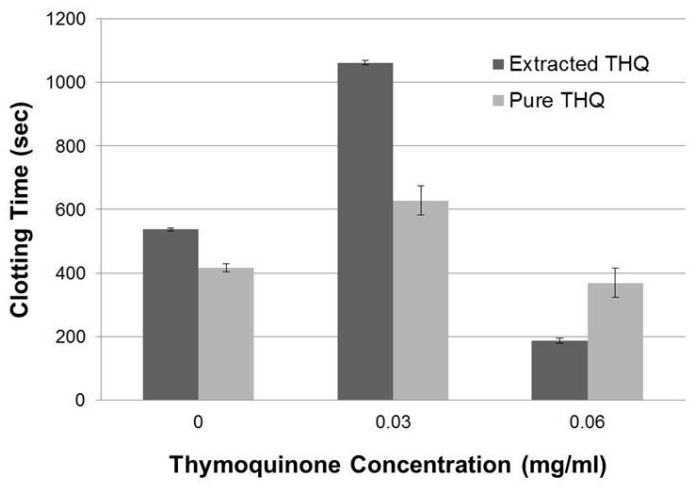
THQ extracted from black seeds and pure THQ exhibit a moderate to slight increase in the basal coagulation time of whole blood. Comparison of the effects of THQ extracted from black seeds and pure THQ on the coagulation time of whole blood measured with TEG assay. Either DMSO or 50% Cremaphore EL:50% EtOH were used as blank controls. Two-way ANOVA test without interaction effects showed that the clotting time is significantly different (F = 9.23; *p* < 0.01) across the THQ concentrations, and that there was no significant difference (F = 0; *p* = 0.51) between extracted THQ and pure THQ. *Post hoc* analysis (Tukey) showed that clotting time for 0.03 mg/mL was significantly greater compared to control (*p* < 0.05) and clotting time was not significantly different for 0.06 mg/mL compared to control. The experiments were performed twice in duplicate. Error bars show the standard deviations of the mean values.

Thymoquinone in the black seed oil extract was tested for its ability to increase the clotting time as measured with TEG assay using normal whole human blood. The clotting time increased approximately two-fold at 0.035 mg/mL of estimated THQ concentration in the black seed oil (Figure 2). However, at higher concentrations the clotting time fell below the control. THQ in the solvent-extracted black seed oil appears to extend the coagulation time of normal human blood.

### 2.2. Pure THQ Modulates Coagulation in Normal Blood and Plasma

Next we tested if purchased THQ (99% purity) exhibited similar effects as extracted THQ from black seeds. The purchased THQ will be referred to as pure THQ. Pure THQ increased the coagulation time of the whole blood at 0.03 mg/mL and did not have any effect at higher concentrations (Figure 2), showing that pure THQ modulates the coagulation of normal human blood within a narrow dose range at lower concentrations. We next tested the effects of pure THQ on the coagulation of normal human plasma by aPTT assay. THQ modestly increased the coagulation time with increasing concentration (Table 1), from basal 37.2 to 50.2 s at the highest concentration tested (1 mg/mL). In comparison, USP heparin increased the coagulation time from basal 36.6 to 219 s at 0.045 mg/mL. Thus, data from both *in vitro* coagulation assays indicate that THQ has a modest effect on the coagulation time of normal human blood and plasma. We used pure THQ for the rest of the study.

### 2.3. Pure THQ Can Reverse TF-Mediated Coagulation on Cancer Cells

**Figure 3:**
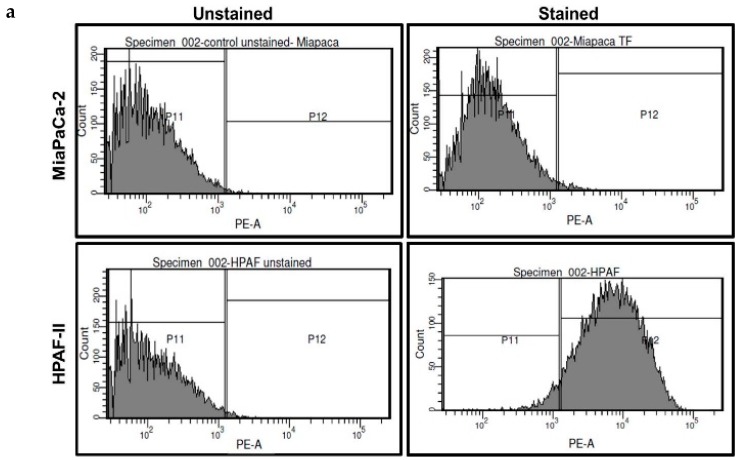

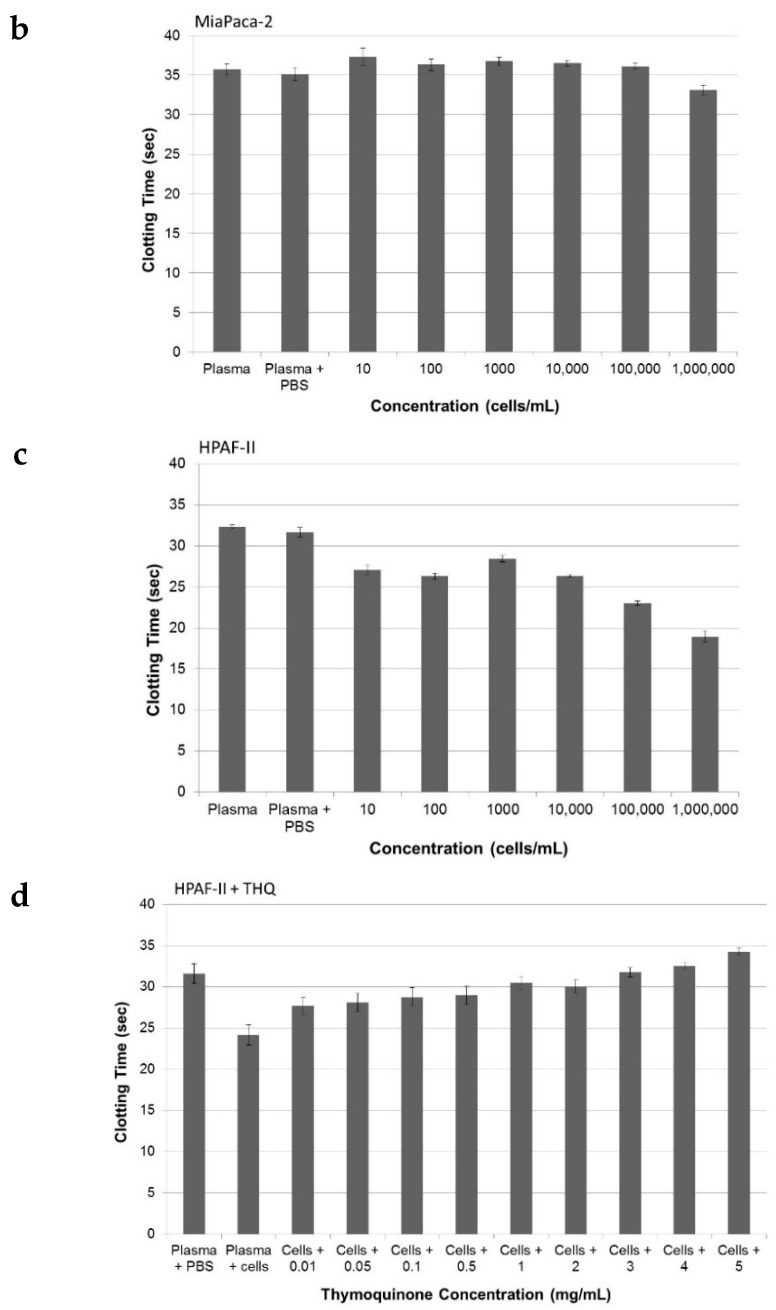
THQ reverses coagulation mediated by tissue factor (TF) present on pancreatic cancer cells. (**a**) Flow cytometry analysis of TF on pancreatic cancer cells. Data in the top panels are from MiaPaCa-2 cells, and data in the bottom panels are from HPAF-II cells. The left panels serve as a control from cells unstained for TF, and the right panels are from cells stained with TF-antibody, followed by PE-conjugated secondary antibody. The top right panel shows the absence of TF on MiaPaCa-2 cells. Presence of TF on HPAF-II cells is confirmed by the TF staining on HPAF-II cells (bottom, right panel). Approximately 98% of HPAF-II cells were positive for the presence of TF; (**b**) MiaPaCa-2 cells do not cause coagulation of plasma. The coagulation time measured with aPTT assay did not show any change in the presence of an increasing number of MiaPaCa-2 cells; (**c**) the coagulation time measured with aPTT assay showed that HPAF-II cells can decrease the coagulation time. Both *t*-test, as well as ANOVA with *post hoc* (Tukey), showed that there was no significant difference in the clotting time between the plasma and plasma with PBS. Addition of cells (10 to 10,000) in each case significantly (*p* < 0.001) reduced the clotting time compared to PBS-plasma; (**d**) THQ reversed the coagulation induced by HPAF-II cells. To 1 × 10^6^ HPAF-II cells, THQ was added in increasing concentration, from 0.01 mg/mL to 5 mg/mL. Both *t*-tests, as well as ANOVA with *post hoc* (Tukey), showed that THQ at each concentration (0.01 to 5 mg/mL) significantly (*p* < 0.001) reduced the clotting time compared to plasma + cells with no THQ. The experiments were performed three times in triplicate. Error bars show the standard deviations of the mean values.

We used two cell lines to test if THQ has the ability to reverse coagulation mediated by cancer cells: MiaPaCa-2 that does not express TF and HPAF-II that expresses TF on its cell surface (Figure 3a). Due to the absence of TF on MiaPaCa-2, increasing the number of these cells did not affect coagulation time as measured with the aPTT assay using human plasma (Figure 3b). However, HPAF-II cells were able to decrease the coagulation time with increasing cell concentration (Figure 3c). The decrease in the coagulation time by HPAF-II cells at every concentration is significant compared to PBS-control (*p* < 0.05). Next, to a constant number of 1 × 10^6^ HPAF-II cells, THQ was added in increasing concentration ranging from 0.01 to 5 mg/mL and aPTT assay was performed. With increasing THQ concentration there was a corresponding extension of the clotting time compared to clotting time mediated by HPAF-II cells alone (Figure 3d). The reversal of coagulation by THQ was significant from the lowest concentration (0.01 mg/mL) tested and can effectively reverse the coagulation mediated by HPAF-II cells to basal levels at 1 mg/mL concentration (Figure 3c).

### 2.4. Pure THQ Can Reverse Coagulation Mediated by Lipopolysaccharide (LPS)

**Figure 4:**
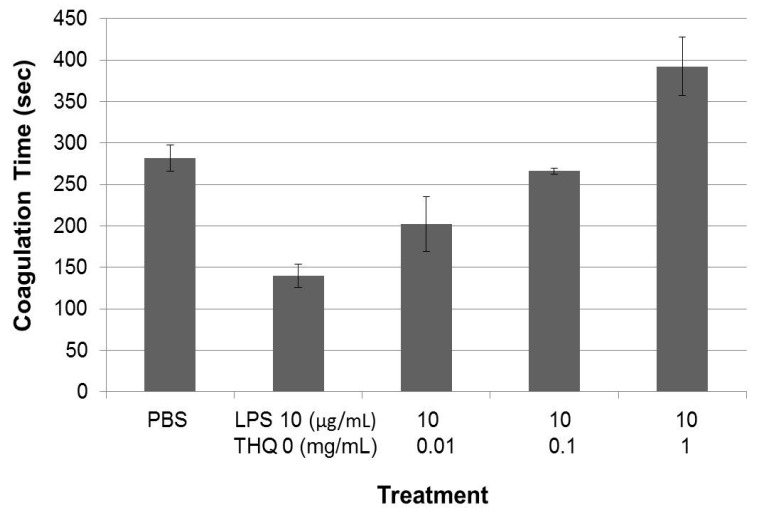
THQ reverses coagulation mediated by lipopolysaccharide (LPS). Increasing concentrations of THQ were pre-incubated with whole blood at 37 °C for 1 h. After the pre-incubation, LPS was added to the blood and incubated for an additional 2 h at 37 °C. The coagulation time of the blood was analyzed with TEG assay. LPS-treatment significantly (*p* < 0.001) decreased the clotting time compared to PBS control, by ANOVA with *post hoc* (Tukey) analysis. Pre-incubation with THQ caused significant (*p* < 0.001) reversal of coagulation mediated by LPS at 0.1 and 1 mg/mL by ANOVA with *post hoc* (Tukey) analysis. Note that the coagulation time is back to that of PBS control at 0.1 mg/mL of THQ concentration. The experiments were performed twice in triplicate. Error bars show the standard deviations of the mean values.

Studies suggest that THQ exhibits its global anti-cancer activities via its effect on inflammatory response [3], and since inflammation also enables cancer-related coagulation [38,39], we tested the effects of THQ on blood coagulation mediated by the inflammatory trigger LPS. Total blood was incubated with LPS at 37 °C for 2 h prior to assessing coagulation time with TEG assay. LPS promoted blood coagulation in a dose-dependent manner, ranging from 0.5 to 10 µg/mL (data not shown). To test if THQ can reverse the LPS-triggered coagulation, an increasing dose of THQ was pre-incubated with blood for one hour at 37 °C prior to addition of LPS at 10 µg/mL. THQ was able to reverse the coagulation triggered by LPS in a dose-dependent manner, restoring the coagulation time triggered by LPS to basal levels at 0.1 mg/mL (Figure 4). Thus, THQ can effectively reverse the coagulation triggered by inflammatory insult and restore the coagulation time to basal levels.

### 2.5. THQ Moderately Inhibits Factor Xa but Not Factor IIa Activity of Coagulation Cascade

**Figure 5:**
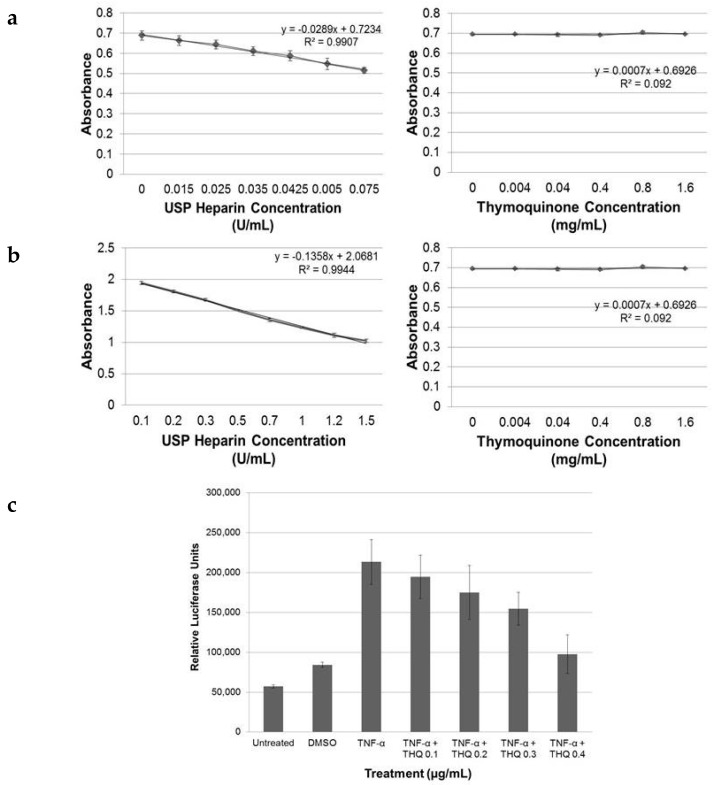
THQ exerts anticoagulant effect by affecting both blood coagulation pathway and inflammatory pathway. (**a**) THQ did not have any effect on factor IIa activity. USP-heparin was used as a positive control; with an increase in concentration of USP-heparin, there was a corresponding decrease in activity of factor IIa (**left** panel). Increasing concentrations of THQ had no effect on the activity of factor IIa (**right** panel); (**b**) THQ moderately decreased factor Xa activity. USP-heparin was used as a positive control; with increasing concentration there was a corresponding decrease in activity of factor Xa (**left** panel). Increasing concentrations of THQ exerted a low to moderate reduction on activity of factor Xa (**right** panel). Note that concentration of USP-heparin is expressed as U/mL and that of THQ is expressed as mg/mL. The concentration of heparin in 0.015 to 0.075 U/mL is 0.00007 to 0.0003 mg/mL. Experiments were performed once with USP-heparin and twice with THQ; (**c**) THQ down-regulates the downstream effects of TNF-α. HeLa cells stably transfected with luciferase gene under the control of NF-κB promoter elements were stimulated with TNF-α at 10 ng/mL. This increased the NF-κB promoter activity four-fold, as shown by luciferase activity. Pre-incubation of HeLa cells with increasing concentrations of THQ, prior to the addition of TNF-α, decreased the TNF-α-triggered-luciferase activity correspondingly, in a dose-dependent manner. ANOVA with *post hoc* (Tukey) showed that THQ at 0.4 mg/mL reduced the TNF-α-induced luciferase activity to basal levels. (*p* < 0.001). The experiments were repeated twice in duplicate. Error bars show the standard deviations of the mean values.

Typically the regulation of coagulation by anticoagulants, such as heparin and its derivatives, occurs by regulating either the activity of factor IIa or factor Xa, or both, of the coagulation cascade [40]. We tested if THQ modulates blood coagulation by having a direct effect on one or both of these factors. Both pure THQ and USP-heparin were tested using an automated chromogenic assay. The inhibitory effects of antithrombin III on factor IIa, factor Xa, and other coagulation serine proteases in plasma are increased several thousand-fold by heparin, accounting for the anticoagulant effect of heparin. In these assays, the inhibition of factor IIa and Xa is directly proportional to the heparin concentration and, therefore, the residual activities of IIa and Xa are inversely proportional to the heparin concentration, which is measured as absorbance at 405 nm. Measurement of the effects of USP-heparin on the inactivation of both factors was done as positive controls (left panels, Figure 5a,b). THQ did not have any inhibitory effect on factor IIa at a concentration between 0.004 and 1.6 mg/mL (right panel, Figure 5a). However, for the same range of concentrations, THQ exhibited inactivation of factor Xa (right panel, Figure 5b). Thus, THQ exerts at higher concentrations (0.8–1.6 mg/mL) a moderate, but significant, inhibitory effect on factor Xa activity and does not exert any inhibitory effect on factor IIa/thrombin activity.

### 2.6. THQ Down-Regulates TNF-α-Mediated Activation of NF-κB

Next, we determined if THQ has direct inhibitory effects on cytokines that promote inflammatory pathways. We used HeLa cells that are stably transfected with NF-κB elements cloned upstream of the luciferase gene. TNF-α is one of the major cytokines that mediates crosstalk between inflammation and thrombosis. We, therefore, used TNF-α (10 ng/mL) to up-regulate the NF-κB promoter elements in HeLa cells. TNF-α treatment increased activity of NF-κB elements by approximately four-fold, as determined with luciferase activity (Figure 5c). To test if THQ can decrease the luciferase activity triggered by TNF-α on the NF-κB promoter elements, we performed luciferase assay after pre-treating the cells with pure THQ for 2 h prior to the addition of TNF-α. The luciferase activity driven by NF-κB promoter elements decreased in correspondence to the increasing dose of THQ from 0.1 to 0.5 µg/mL (Figure 5c). However, treatment with THQ alone did not have any effect on the NF-κB-mediated luciferase activity (data not shown). THQ could effectively inhibit the downstream activities of TNF-α on the NF-κB promoter.

## 3. Discussion

We have shown, for the first time, that THQ, both extracted from black seeds and its pure form, has the ability to modulate blood coagulation *in vitro*. We have confirmed via mechanistic studies that THQ interferes with the blood coagulation by directly decreasing, weakly, but significantly, the activity of factor Xa in blood coagulation pathway and by down-regulating the downstream effects of TNF-α, a cytokine that plays a critical role in the crosstalk between the inflammatory pathway and thrombosis. 

**Figure 6:**
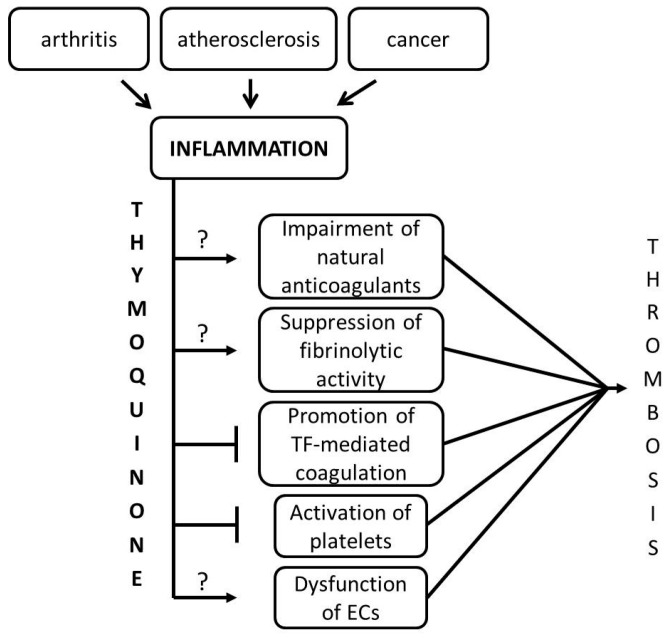
THQ interferes with the crosstalk between inflammation and thrombosis. Inflammation is triggered under various pathophysiological conditions in the body such as arthritis, atherosclerosis, and cancer. An increase in blood coagulation or thrombosis is seen in all of these conditions. Inflammation-triggered cytokines, particularly TNF-α, IL-1, and IL-6, enable a cascade of events, culminating in thrombosis. We provide evidence in this study that THQ can effectively inhibit tissue factor (TF)-mediated coagulation and inactivation of platelets that lie in the crosstalk between inflammation and thrombosis. The effect of THQ on natural anticoagulants, endothelial cells (ECs), and fibrinolytic activity could not be investigated in this study due to limitations of the *in vitro* system; this is represented by ‘?’. In addition, THQ also exerts an effect, although moderate, on the factor Xa activity of the blood coagulation system.

Inflammation and hemostasis are two physiologic processes that have significant influence on each other, in that inflammation leads to activation of hemostasis which, in turn, influences inflammation [41]. During an inflammation response, pro-inflammatory cytokines, such as TNF-α, IL-1, and IL-6, exhibit a critical effect on the hemostatic system [42]. Pathological conditions in which the regulatory relationship between inflammation and thrombosis contribute to the progression of the severity of the diseases are atherosclerosis, cancer-related thrombosis, and sepsis-induced thrombosis. Inflammatory mediators shifts the hemostatic balance to a pro-coagulant/thrombotic state by altering the endothelial cell function, affecting platelet activation, interfering with TF-mediated coagulation, impairing the function of natural anticoagulants, such as Protein C, Tissue factor pathway inhibitor (TFPI), antithrombin, and by suppressing the fibrinolytic activity (Figure 6) [41].

In our study, TEG data shows that black seed oil containing THQ has the capacity to increase the coagulation time by approximately two-fold only at 0.035 mg/mL without any dose-dependent increase in coagulation time, while pure THQ can moderately increase the coagulation time at 0.03 mg/mL. In addition, with aPTT assay, we found that THQ can moderately increase the coagulation time of normal human plasma in a dose-dependent manner. The increase of only ~13 s is minimal in that the coagulation time was extended by the highest concentration of THQ tested (1 mg/mL). This is in stark contrast to the 182 s increase by USP-heparin at 0.045 mg/mL. Thus, THQ does not affect the coagulation time of normal blood or plasma, even at higher concentration. 

Malignant cells activate blood coagulation by releasing cytokines that interact with endothelial cells and other blood cells to trigger inflammation by the release of pro-inflammatory cytokines and by producing TF either on their cell surface or by releasing tiny particles, called microparticles, with TF on their surface [35,43]. The negative charge on the TF enables the assembly of different proteins of the coagulation cascade, and indeed the levels of TF are increased in cancer patients diagnosed with venous thromboembolism (VTE) compared to cancer patients without VTE [44]. Since the aPTT assay utilizes human plasma devoid of blood cells, it can be concluded that the anticoagulant effect of THQ interference on TF-mediated coagulation shown here is due to direct effects of THQ on TF present on the cancer cells. Furthermore, THQ inactivated activation of factor Xa, indicating that THQ has a direct effect on the activation of fibrinogen to form fibrin clots. THQ also had a significant effect on the reversal of coagulation mediated by LPS as measured with the TEG assay, providing indirect evidence that THQ can interfere with platelet function in enabling coagulation. The TEG assay utilizes whole blood and, thus, the direct effect of THQ specifically on platelets cannot be analyzed due to the presence of other blood cells such as monocytes and neutrophils, which are known to release pro-inflammatory cytokines, such as TNF-α that promote thrombosis.

It is clear that pure THQ has only a minimal and a modulatory effect on the coagulation of normal blood and plasma within a narrow dose range at lower concentration, and with no effect at higher concentration of THQ on blood coagulation. This is in contrast to its significant effects on the reversal of blood coagulation triggered by TF and LPS, even at lower concentrations of 0.01 mg/mL. THQ also restored the coagulation time to basal levels in the above conditions. This data suggests that THQ is a suitable candidate and has the potential to be utilized both as an alternative to preventative anticoagulants and as a supplement to the existing therapies for infections, chemotherapies, and anticoagulant therapies.

Data from the luciferase assay indicated that THQ has direct effects on suppressing the downstream effects of TNF-α. However, this does not mean that THQ would exhibit similar effects on IL-1 and IL-6, other cytokines that promote thrombosis. Thus, THQ interferes at the crosstalk of inflammation and thrombosis specifically in the promotion of TF-mediated coagulation and in the inactivation of platelets (Figure 6). Here, however, we have not studied the effects of THQ on other mediators due to limitations of the *in vitro* system. Having confirmed the anticoagulant effects of THQ in blood coagulation, future studies will be on the *in vivo* effects of THQ in other inflammatory-mediated processes culminating in thrombosis.

This study confirms that THQ has minimal effect on normal blood coagulation and can effectively reverse sepsis-mediated and cancer-mediated thrombosis. This provides a proof-of-concept and points to pursuit of *in vivo* mechanistic, pharmacokinetic and pharmacodynmaic investigations to evaluate the potential use of the natural supplement THQ as an alternative to preventative anticoagulants and/or for use as a supplement to existing therapies for infections, chemotherapies, and anticoagulant therapies.

## 4. Materials and Methods

### 4.1. Materials

All cell culture reagents including media (DMEM, RPMI, EMEM), penicillin, streptomycin, FBS, l-glutamine, hygromycin B, along with pure THQ, Methyl-T-Butyl-Ethyl (MBE), Cremaphore-l, EtOH, and LPS were purchased from Sigma (St. Louis, MO, USA). Black seeds were purchased from a local herbal grocery store in Albany, NY, USA. USP-heparin reference standard was purchased from U.S. Pharmacopeia (Rockville, MD, USA).

### 4.2. Blood Sampling

Blood was drawn from consenting volunteers under a protocol approved by the Institutional Review Board of Albany College of Pharmacy and Health Sciences. Blood was drawn using a BD Vacutainer blood collection set (BD Biosciences, San Jose, CA, USA) into BD Vacutainers, plus plastic citrate (3.2%) blood collection tubes. Thrombelastography (TEG) assay was performed within 3 h of blood collection.

### 4.3. Cell Culture

MiaPaCa-2 cells were obtained from Dr. Thiruvengadam Arumugam (MD Anderson Cancer Center, Houston, TX, USA) and grown in DMEM. HPAF-II cells were purchased from ATCC (Manassas, VA, USA) and grown in EMEM media. HeLa-NF-κB luciferase cells were purchased from Signosis Inc. (Santa Clara, CA, USA) and were grown in DMEM supplemented with hygromycin B (50 µg/mL). All the cells were maintained in their respective media supplemented with 10% FBS, 2 mM l-glutamine, penicillin (100 U/mL), and streptomycin (10 mg/mL) and maintained at 37 °C in a 5% CO_2_ humidified incubator.

### 4.4. Flow Cytometry

One million MiaPaCa-2 and HPAF-II cells were surface-stained for the presence of TF using rabbit polyclonal antibody to TF (Abcam, Cambridge, MA, USA) in 100 µL PBS-5% BSA at 1:100 dilution. The cells were washed and stained with rabbit secondary antibody conjugated with PE and analyzed by flow cytometry. Flow cytometry analysis of the stained cells was performed at the NeuraCell Core Laboratory, located at the Neural Stem Cell Institute (Rensselaer, NY, USA) using FACSAria II (BD Biosciences, San Jose, CA, USA).

### 4.5. Luciferase Assay

Pure THQ powder was dissolved in DMSO to obtain a 10 mg/mL stock solution and diluted in PBS (instead of 50% Cremaphore-EL: 50% EtOH, which we found decreased the cell viability). HeLa cells (1 × 10^5^) were plated into each well in a 12-well dish. The next day, the media was changed to media containing 0.5% FBS and cells were pre-treated with THQ at various concentrations (0.1, 0.2, 0.3, 0.4, and 0.5 µg/mL). After 2 h, TNF-α was added at 10 ng/mL concentration and cells were incubated overnight at 37 °C. The next day, the cells were lysed in 200 μL of CCLR lysis buffer (Promega, Madison, WI, USA). The luciferase activity of the lysates was measured as per manufacturer’s instructions, using a manual luminometer.

### 4.6. Extraction and Estimation of Amount of THQ in Black Seeds

Black seeds (28.5 g) were ground and suspended in 125 mL of MBE. The suspension was placed on a shaker for 3 h, and kept at room temperature. After 24 h, 100 mL of water was added and the suspension was placed on a shaker for 2 h. The solution was then centrifuged at 1200× *g* for 15 min and dried under N_2_. Approximately 8.5 mL of slightly viscous oil was obtained (30 mL/100 g of black seeds) and appeared straw-yellow in color. The aqueous phase was centrifuged at 1200× *g* for 15 min and the supernatant yellow-colored liquid was filtered to remove black solid floccules. The filtered solution was lyophilized to obtain 2.5 g solid substance. 

The amount of THQ in black seed extract was determined with an HPLC method. The chromatographic system consisted of a Waters 2695 Alliance Separations Module (Milford, MA, USA) with a column oven and a 2996 photo diode array detector. A Waters Sunfire^TM^, C_18_, 3.5 µm, 3.9 × 150 cm analytical column was used for separation of THQ at 40 °C and eluted with a gradient of 65% to 90% methanol containing 0.1% formic acid over 5 min at a flow rate of 0.4 mL/min. The UV detector wavelength was 254 nm. The total analysis cycle time was 8 min. A calibration curve was made from standard solutions of THQ to estimate the amount of THQ in the black seed extract.

### 4.7. Activated Partial Thromboplastin Time (aPTT) Assay

The aPTT assay was performed using fresh frozen single donor human plasma obtained and processed through standard methods with prior consent from the donors. Frozen plasma was thawed and allowed to reach room temperature before performing the assay. Pure THQ powder was dissolved in 50% Cremaphore EL: 50% EtOH to obtain a 10 mg/mL stock solution and dilutions were made in PBS. For each assay, 300 µL of plasma was aliquoted and mixed with 30 µL of sample (diluted THQ with or without cells). The assay was performed using the ACL 8000 coagulation analyzer (Beckman Coulter Inc., Lexington, MA, USA) as per the manufacturer’s instructions. Results were measured in seconds with a clot-based method. All assays were repeated twice in triplicate.

### 4.8. Thrombelastography (TEG) Assay

Fresh blood samples were maintained on a rotor with constant mixing. Black seed oil was diluted in DMSO to obtain various concentrations of THQ to perform the TEG assay. THQ concentrations were determined with HPLC. Pure THQ powder was dissolved in 50% Cremaphore EL:50% EtOH to obtain a 10 mg/mL stock solution; further dilutions were made in PBS. For each assay, 340 µL of blood was aliquoted with 20 µL of 0.2 M calcium chloride. The volume of the reagents tested was maintained at 40 µL and assayed using the TEG Hemostasis Analyzer System (Model 5000, Haemoscope Corporation, Niles, IL, USA) per the manufacturer’s instructions. TEG parameters investigated were R-Time (clotting time in min) and maximum amplitude (clot strength in millimeters) through a torsion and pin method of clotting as previously reported [45]. All assays were repeated twice in duplicate or triplicate.

### 4.9. Factor IIa/Xa Assays

The activity of the coagulation factors was determined in the presence of heparin and THQ, using chromogenic procedures [46]. Pure THQ powder was dissolved in 50% Cremaphore EL:50% EtOH to obtain a 10 mg/mL stock solution and dilutions were made in PBS. Activity of factor IIa was determined using an ACTICHROME^®^ Heparin (Anti-FIIa) kit from Sekisui Diagnostics (Stamford, CT, USA), and factor Xa activity was measured using a kit (item no. 0020009400) from Instrumentation Laboratories (Bedford, MA, USA). The absorbance was obtained using the ACL 8000 coagulation analyzer at 405 nm as per the manufacturer’s instructions.

### 4.10. Statistics

Results are presented as the means ± standard deviation comparing experimental and control groups. *T-*test and one-way analysis of variance (ANOVA) were used for statistical analyses, and results were considered statistically significant if *p* < 0.05. Two-way ANOVA with *post hoc* analysis was performed using STATA.

## Figures and Tables

**Table 1:** Comparison of coagulation times between USP heparin and Thymoquinone (THQ) by aPTT assay.

Final Concentration (mg/mL)	Heparin avg. Coagulation T (Sec)	Standard Deviation	THQ avg. Coagulation T (Sec)	Standard Deviation	*T*-test *p* values
0.000	36.567	0.513	38.200	0.000	
0.010	53.967	0.751	38.400	0.361	
0.015	64.500	0.693			
0.020	81.667	0.404			
0.025	109.333	0.577			
0.030	123.000	10.536			
0.035	148.667	1.528			
0.040	184.333	2.517			
0.045	219.000	2.000			
0.050			38.567	0.603	
0.100			39.800	1.153	
0.200			42.800	0.854	0.004
0.300			43.833	1.041	0.004
0.400			44.067	0.902	0.002
0.500			47.667	1.258	0.002
1.000			50.233	3.044	0.009

## Abbreviations

aPTT	activated partial thromboplastic time
LPS	lipopolysaccharide
TF	tissue factor
TEG	thrombelastography
THQ	Thymoquinone
VTE	venous thromboembolism
